# Examining drivers of self‐harm guideline implementation by general practitioners: A qualitative analysis using the theoretical domains framework

**DOI:** 10.1111/bjhp.12598

**Published:** 2022-04-13

**Authors:** Jessica Z. Leather, Christopher Keyworth, Nav Kapur, Stephen M. Campbell, Christopher J. Armitage

**Affiliations:** ^1^ NIHR Greater Manchester Patient Safety Translational Research Centre The University of Manchester Manchester Academic Health Science Centre Manchester UK; ^2^ Manchester Centre for Health Psychology Division of Psychology and Mental Health School of Health Sciences University of Manchester Manchester UK; ^3^ The School of Psychology The University of Leeds Woodhouse, Leeds UK; ^4^ Centre for Mental Health and Safety University of Manchester Manchester Academic Health Science Centre Manchester UK; ^5^ Greater Manchester Mental Health NHS Foundation Trust Manchester Academic Health Science Centre Manchester UK; ^6^ Centre for Primary Care and Health Services Research School of Health Sciences University of Manchester Manchester UK; ^7^ Manchester University NHS Foundation Trust Manchester Academic Health Science Centre Manchester UK; ^8^ NIHR Manchester Biomedical Research Centre Manchester University NHS Foundation Trust Manchester Academic Health Science Centre The Nowgen Centre Manchester UK

**Keywords:** evidence‐based guidelines, general practice, self‐harm

## Abstract

**Objectives:**

This study aimed to (1) examine barriers and enablers to General Practitioners’ (GP) use of National Institute for Health and Care Excellence (NICE) guidelines for self‐harm and (2) recommend potential intervention strategies to improve implementation of them in primary care. Design: Qualitative interview study.

**Methods:**

Twenty‐one telephone interviews, semi‐structured around the capabilities, opportunities and motivations model of behaviour change (COM‐B), were conducted with GPs in the United Kingdom. The Theoretical Domains Framework was employed as an analytical framework. Using the Behaviour Change Wheel, Behaviour Change Techniques (BCTs), intervention functions and exemplar interventions were identified.

**Results:**

GPs valued additional knowledge about self‐harm risk assessments (knowledge), and communication skills were considered to be fundamental to high‐pressure consultations (cognitive and interpersonal skills). GPs did not engage with the guidelines due to concerns that they would be a distraction from patient cues about risk during consultations (memory, attention and decision processes), and perceptions that following the guidance is difficult due to time pressures and lack of access to mental health referrals (environmental context and resources). Clinical uncertainty surrounding longer term care for people that self‐harm, particularly patients that are waiting for or cannot access a referral, drives GPs to rely on their professional judgement over the guidance (beliefs about capabilities).

**Conclusions:**

Three key drivers related to information and skill needs, guideline engagement and clinical uncertainty need to be addressed to support GPs to be able to assess and manage self‐harm. Five intervention functions and ten BCT groups were identified as potential avenues for intervention design.

**Statement of contribution**

***What is already known on this subject?***

- General Practitioners (GPs) have unique opportunities to identify and intervene in self‐harm.
- Some GPs do not implement NICE guidance about self‐harm due to a lack of awareness and resources.
- The drivers of guideline use that could potentially inform intervention strategies are unclear.

***What does this study add?***

- GPs need further training to address skill gaps relating to consultations about self‐harm.
- Guidelines must be optimised for quick‐reference to support decision‐making during consultations.
- Further guidance is needed about supporting patients who self‐harm while waiting for a referral.

## INTRODUCTION

Self‐harm encompasses ‘any act of self‐poisoning or self‐injury carried out by an individual irrespective of motivation’ (National Institute for Health & Care Excellence, 2011), p. 4 and is an important risk factor for suicide among adults of all ages (Bergen et al., 2012). Services for self‐harm in the United Kingdom (UK) are under intense demand and scrutiny due to the increased incidence of self‐harm following the 2008 economic recession (McManus et al., 2019). Primary care is a key setting for assessing and managing self‐harm, since many people who self‐harm do not present to mental health services (Geulayov et al., 2016). General practitioners (GPs) are at the frontline of health care, which provides them with unique opportunities to identify and intervene in self‐harm in a less stigmatizing environment than secondary care or emergency services (Centre for Mental Health, 2019). However, self‐harm often occurs in the context of complex mental health issues such as adverse childhood experiences (Fliege et al., 2009), trauma (Barnicot & Crawford, 2018) and personality disorders (Witt et al., 2019); such predictors of self‐harm present considerable challenges for GPs meaning self‐harm behaviours are rarely addressed in isolation from their underlying issues (Mughal et al., 2020). While traditionally GPs have been perceived as gatekeepers to specialist services (Saini et al., 2016), they are under considerable pressure to manage self‐harm within primary care and advocate for patients to pursue self‐help (Bailey et al., 2017; Mughal et al., 2020). Although data suggest the incidence of primary care‐recorded self‐harm fell during the initial wave of COVID‐19 in the United Kingdom (Carr et al., 2021; Kapur et al., 2021), there are concerns that services will face an increase in demand for mental health‐related concerns after further periods of national lockdown (Mughal et al., 2021; Williams et al., 2020).

The NICE guidelines for the management of self‐harm (National Institute for Health & Care Excellence, 2004, 2011) contain recommendations about treatment and referral options to support healthcare professionals to provide the best care for patients. However, a survey of 200 GPs found 45% reported never using NICE guidelines for self‐harm, instead preferring to rely on intuition when encountering a patient who has self‐harmed; of the GPs that were aware of the guidelines, 38% perceived them to be useful (Cello Health PLC, 2012). A recent representative survey of 67 GPs found only 36% (*n* = 24) were knowledgeable about the self‐harm guidelines and implemented the guidance with just 62% of the patients they encountered that had self‐harmed or were at risk of repeat self‐harm (Leather et al., 2020). GPs have long cited difficulties when attempting to arrange referrals in accordance with guidelines (Prasad et al., 1999). More recent studies have reported that GPs feel alienated from secondary services, which can be challenging to reach (Wand et al., 2018). Often a GP’s ability to follow the recommendations made for them in the guidance is hampered by barriers inherent to general practice, such as time constraints, appointment availability and a systemic lack of access to secondary mental health teams (Centre for Mental Health, 2019; Mughal et al., 2020). GPs experience training about mental health which includes awareness of evidence‐based guidelines (Royal College of General Practitioners, 2019); however, acquiring knowledge about self‐harm and the self‐harm guidelines is not a mandatory part of core GP training. Systematic review evidence suggests that prior clinical experiences, including the uptake of further training, also influence GPs’ use of guidelines (Zwolsman et al., 2012); trainee GPs encounter more clinical uncertainty (Chatterjee et al., 2017; Welink et al., 2020) and consult guidelines more readily than experienced GPs who are confident making decisions based upon their expertise (Francke et al., 2008; Harris et al., 2014; Patel et al., 2001; Van Dijk et al., 2010). There is a mounting patient safety rationale to ensure evidence‐based guidelines for self‐harm are implemented (Carr et al., 2016).

Although GPs are used to managing general mental health issues as part of their role (Saini et al., 2010), the quality of this management varies (Gask et al., 2008; Menear et al., 2014). Detection of common mental health conditions is low in general practice due to missed opportunities for screening (Mitchell et al., 2009). Treatments tend to be focused towards pharmacological options, which are not always provided in accordance with national guidance (Tobin et al., 2020; Toner et al., 2010), and communication difficulties can deter patients from seeking further help or complying with their treatments (Ford et al., 2019; Salmon et al., 2004). Many GPs feel ill‐equipped to handle self‐harm and prevent potential suicide attempts among their patients (Chandler et al., 2016). Lack of confidence can be exacerbated by the additional difficulties that can accompany patients who self‐harm, such as complex mental health history (Fliege et al., 2009; Witt et al., 2019), non‐attendance (Neeleman et al., 2009; Williams et al., 2020) and frequent, lengthy consultations (Bailey et al., 2019). Australian GPs described feeling impotent and hopeless about managing the complex underlying factors contributing to self‐harm in elderly patients (Wand et al., 2018). Similarly, a survey of 28 GPs in the United Kingdom reported that they felt under‐skilled or lacked training to talk about self‐harm, resulting in them missing opportunities to identify self‐harm in young people (Fox et al., 2015). A further study found GPs also require confidence and environmental enablers such as extended appointment times to be able to broach the topic of self‐harm (Bailey et al., 2017). Beyond skills and confidence, emotional factors such as empathy have also been found to mediate the association between perceived knowledge and attitudes about self‐harm (Moriarty et al., 2020). While these studies have identified salient barriers that inhibit or prevent guideline implementation, they lack a strong theoretical basis; as a result, they are unlikely to have captured a comprehensive range of drivers. Since guideline implementation is considered a form of behaviour change (Heslehurst et al., 2014) implementation strategies require an in‐depth understanding of the complex determinants of healthcare professional behaviour, informed by behaviour change theory, to identify causal processes and relevant behaviour change techniques (BCTs) (Michie et al., 2005).

The Theoretical Domains Framework (TDF) integrates numerous theories of behaviour change into a single, comprehensive framework to encapsulate cognitive, affective, social and environmental influences on professional practice (Atkins et al., 2017). It was designed and validated for use in implementation research to increase the accessibility and utility of psychological theory for understanding behaviour change and intervention design (Cane et al., 2012; Michie et al., 2005). An expert consensus group synthesized constructs from existing behaviour change theories relevant to healthcare professional behaviours into 14 domains. These domains expand upon the components of the capabilities (C), opportunities (O) and motivations (M) model of behaviour change (B; COM‐B) (Michie et al., 2011), a model comprising six components that drive behaviour: physical capability (e.g. skills), psychological capability (e.g. knowledge), physical opportunity (e.g. time), social opportunity (e.g. social cues), automatic motivation (e.g. emotional reactions) and reflective motivation (e.g. intentions) (Michie et al., 2014). For example, the COM‐B component ‘psychological capability’ maps to the ‘knowledge’, ‘skills’, ‘memory attention and decision processes’ and ‘behavioural regulation’ domains of the TDF, which demonstrates the finer grain of detail the TDF offers about behavioural drivers (Cane et al., 2012). In summary, the TDF and COM‐B offer a systematic approach to identify barriers and enablers of evidence‐based practice, which allows for a theory‐based development of interventions by selecting appropriate BCTs, intervention functions and policy categories that correspond to each domain (Cane et al., 2015; Michie et al., 2014).

Interventions are required to empower GPs to assess and manage self‐harm appropriately and feasibly, to increase implementation of national guidelines and address the gaps in self‐harm prevention accentuated by the COVID‐19 pandemic (Mughal et al., 2021). Although a number of studies have identified several barriers and enablers to effective self‐harm practice, there has yet to be a theoretically grounded, comprehensive investigation into drivers of national guideline implementation for self‐harm among GPs. Our study addresses this gap for the first time by using the TDF as an analytical framework to identify salient drivers and examine how these drivers could be used to inform the development of an intervention to support self‐harm guideline implementation in primary care (Atkins et al., 2017; Cane et al., 2012). The TDF has been used elsewhere to develop interventions for GPs’ clinical practice, including reducing imaging for low back pain (Jenkins et al., 2018) and improving medicine management for people with dementia (Barry et al., 2020); the COM‐B has been used to capture the barriers and enablers to the implementation of clinical guidelines (e.g. (Bailey et al., 2019; Fox et al., 2015)). This suggests the TDF is an appropriate framework with which to explore this area of professional practice. The purpose of the present study was to (1) examine the barriers and enablers to GP’s use of, and adherence to, the NICE guidelines for self‐harm, and (2) recommend potential intervention strategies to improve implementation of the NICE guidelines in primary care.

## METHODS

### Philosophical stance

The research was conducted under a pragmatic paradigm; an action‐focused perspective that aims to interpret knowledge in a manner that produces functional consequences (Cornish & Gillespie, 2009). Therefore, the ontological stance of this research is that reality and knowledge are socially constructed and encountered through interpreting human experience (Kaushik & Walsh, 2019). Our rationale for this approach is to translate knowledge about healthcare professional behaviour into intervention targets.

### Participants

Twenty‐one GPs working in the United Kingdom were purposively invited to take part in an interview study. Participants were previously recruited through a survey panel company (YouGov) to take part in a cross‐sectional survey examining implementation of the NICE guidelines for self‐harm among a large, representative sample of UK healthcare professionals (Leather et al., 2020). Sixty‐one GPs from that sample who had heard of the NICE guidelines for self‐harm were invited to participate in follow‐up interviews, of which 21 agreed to take part. A sample quota of 22 was set, but data collection ceased when the research team agreed that no new themes were emerging from the data suggesting saturation had been reached (Guest et al., 2006). No novel data were generated from the final few interviews, which suggests data saturation was achieved.

### Design

General practitioners working in the United Kingdom were interviewed by telephone using a semi‐structured topic guide. The guide (Appendix S1) was adapted from an existing schedule (Keyworth et al., 2019) and was based on the COM‐B (Michie et al., 2011). Using the COM‐B as a basis for the interview questions allowed us to (a) explore the barriers and enablers to implementing the NICE guidelines for self‐harm, (b) use the TDF as an analytical framework to categorize themes generated from the data and (c) link the components of the COM‐B model to the TDF framework to specify the barriers and enablers to implementation of the NICE guidelines for self‐harm in general practice.

The Behaviour Change Wheel (Michie et al., 2014) was used to interpret the theoretical domains and identify functions and BCTs to illustrate how a behaviour change intervention could target each domain (Cane et al., 2015). The Behaviour Change Wheel is an amalgamation of nineteen frameworks of behaviour change interventions and uses the COM‐B as its central hub. It contains nine categories of intervention functions to address deficiencies in capabilities, opportunities or motivations (e.g. Enablement), and seven policy categories that could enable those interventions (e.g. Legislation). We provide examples of operationalized BCTs and intervention functions to demonstrate how they could be used to improve GPs’ implementation of the NICE guidelines for self‐harm.

### Procedure

A university ethics committee granted ethical approval in February 2019 (Ref: 2019–5456–9504). A topic guide and accompanying information sheet (Appendix S2) were developed for the panel company's interviewers to refer to. The interviewers were conducted by two members of the panel company (one male), who were trained in qualitative interviewing. No prior relationship was established between the participants and interviewers. Utilizing interviewers external to the research team may reduce the risk of researcher bias in data collection (Crilly et al., 2020; Jorgenson et al., 2012). The interviewers were instructed to (a) use open‐ended questions to facilitate exploration of barriers and enablers of guideline implementation; (b) use caution when asking about current practice to minimize social desirability or professional identity bias; and (c) ask for specific examples of current practice where they encountered a patient who had self‐harmed (Michie et al., 2014).

After completion of an online survey (Leather et al., 2020), participants were invited to take part in the interview study by the panel company and were incentivized with a points‐based reward system (YouGov, 2018). Interviews were audio‐recorded and transcribed verbatim, then anonymized and transferred to the research team for analysis. Informed consent was obtained before each interview. In accordance with YouGov's GDPR regulations, no personally identifiable participant data were shared with the research team. Data collection took place between April 2019 and May 2019.

### Analysis

A combination of content analysis and framework analysis was used to analyse the data. Microsoft Excel was used to develop the coding framework. Two members of the research team (JZL and CK) analysed half of the interviews each. Both parties checked each other's coding during ongoing data analysis meetings, and unanimous agreement was reached for codes assigned to the data. JZL then matched data to the domains, and CK reviewed matching for the first 25% of the interviews assigned to the framework. Good agreement (>60%; (Mitchell et al., 2009)) was achieved, and remaining discrepancies were resolved through discussion to ensure an appropriate domain was agreed upon. This ensured the coding and mapping process was consistent across coders.

Two levels of coding were used. Deductive (first level) coding was used initially to generate the coding framework. Instances of the TDF domains in the data were identified and categorized using directed content analysis, by recording any occurrences relating to TDF domains in the transcripts (Hsieh & Shannon, 2005; Ritchie et al., 1994). A framework approach (Gale et al., 2013) was used to map the data onto relevant domains of the TDF. This allowed the researchers to identify predetermined and emergent issues in the data and use the TDF as an explanatory framework. Occurrences of COM‐B components were coded and mapped directly on to the relevant TDF domains (as specified in (Keyworth et al., 2019)). Salient domains were selected based on two criteria, which have been used in previous research (Gould et al., 2018; Keyworth et al., 2019): (1) domains mentioned by more than 60% of participants, and (2) strong importance expressed spontaneously by participants. Key domains met both criteria. Inductive (second level) coding was done by generating explanatory themes for the key theoretical domains identified in the first‐level coding (Atkins et al., 2017). Finally, relevant BCTs were mapped to each TDF domain to illustrate how the findings could be used to inform intervention design (Cane et al., 2015).

## RESULTS

**FIGURE 1:**
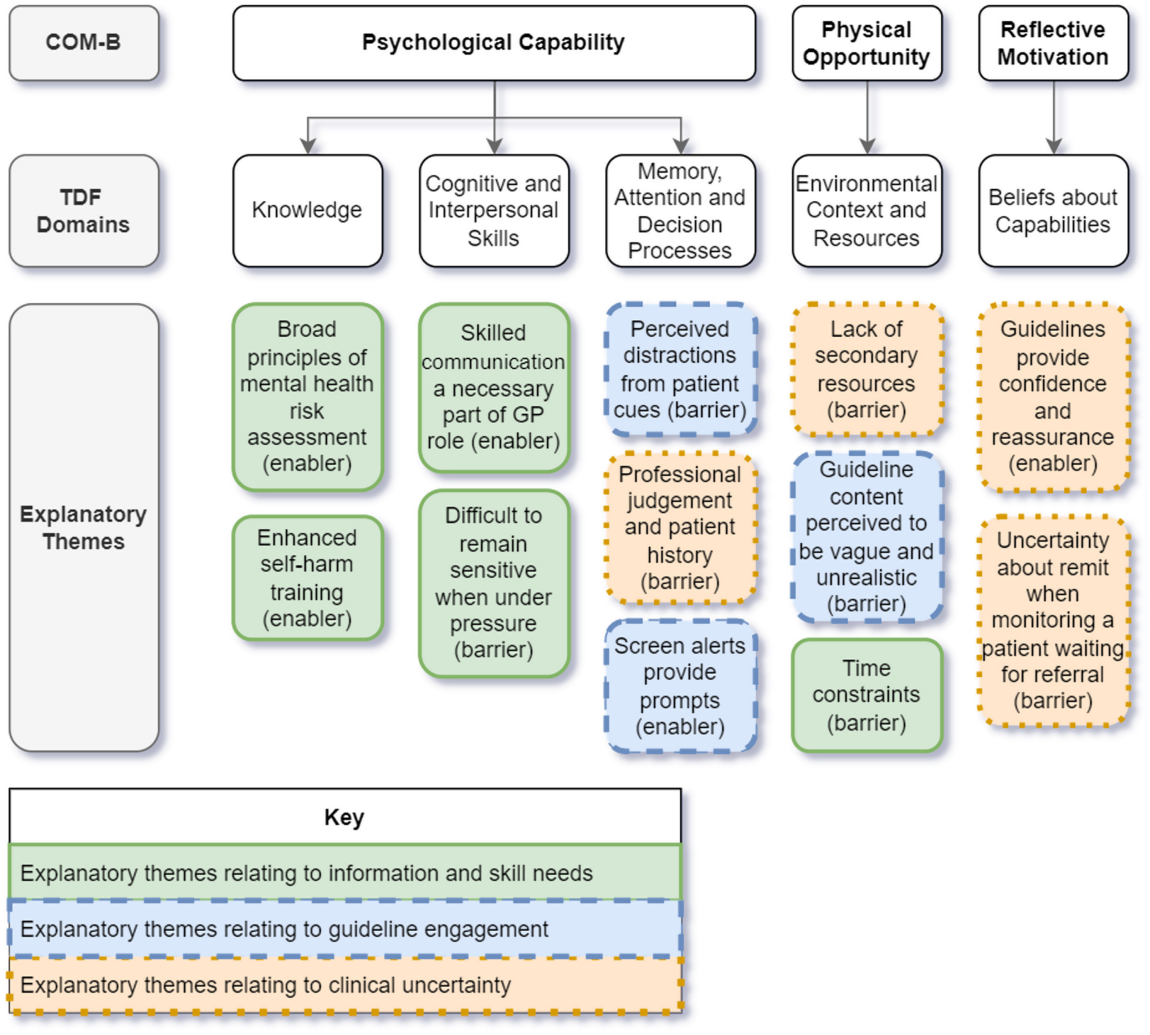
Barriers and enablers to implementing the NICE guidelines for self‐harm

Participants were UK general practitioners working in NHS GP surgeries. Demographics are presented in Table 1. Length of interviews ranged from 18:22–65:00 min (mean length 29 min). Results are presented in terms of theoretical domains and explanatory themes. There were no substantive differences in interview responses by gender or age. A diagram illustrates key findings in Figure 1, and a summary table is presented in Table 2.

**TABLE 1:** Participant demographics (*n* = 21)

Variables	*N* (%)
Gender	
Male	3 (14.29)
Female	16 (76.19)
Did not state	2 (9.52)
Age	
25–34	11 (52.38)
35–44	4 (19.05)
45–54	1 (4.76)
55–64	1 (4.76)
Did not state	4 (19.05)
Years in profession	
Still qualifying/first year	6 (28.57)
1–3 years	3 (14.29)
4–6 years	7 (33.33)
7–10 years	4 (19.05)
Over 20 years	1 (4.76)
GP role	
Trainee	6 (28.57)
Locum	7 (33.33)
Salaried	5 (23.81)
Partner	3 (14.29)

**TABLE 2:** Summary of key findings

COM domain	TDF domain	Description of domain	Exemplar quotes	Intervention function	Individual BCTs	Exemplar interventions
Psychological capability	Knowledge	GP mental health training focuses broadly on risk assessment. Further self‐harm training is beneficial.	‘Case‐based training or even speaking to the patient who's self‐harmed in the past and finding out, kind of, what happened to them and what doctors did to improve. But it would be great if there [was a]… set protocol on what to look out for, what the red flags were and different steps’ (Trainee GP 11)	Education	Information about antecedents Prompts/cues Information about health consequences	Patient‐led information about risk cues for self‐harm, to prompt GPs to assess risk of self‐harm or suicide using specific questions (IF: Education; BCT: Prompts/cues).
	Cognitive and interpersonal skills	Communication skills a necessary part of GP’s role to gather information and make decisions. Concerns about managing distressed patients.	‘You have your standard questions that you know you want to ask, and you work those into the consultation, but you do that with a natural flow… If you're feeling low in yourself, truly low, the last thing that you want to do… is go through a prescriptive questionnaire and act like you're speaking to somebody in a call centre’. (Trainee GP 20)	Training	Instruction on how to perform a behaviour Behavioural rehearsal/practice Habit formation Self‐monitoring outcomes of behaviour	Prompt GPs to practice de‐escalating a distressed consultation through role‐play exercises with colleagues (IF: Training; BCT: Behavioural rehearsal/practice).
	Memory, attention and decision processes	Strictly following guidance can distract attention from the patient; gut feeling can supersede guidelines. Screen alerts may prompt engagement with guidelines.	‘When you're doing risk assessments… just those few seconds away where you're looking at the computer or typing, you miss moments with the patient where they might open up or you just miss the odd little slip in their body language that might help you’. (Locum GP 6)	Training Environmental restructuring Enablement	Self‐monitoring outcomes of behaviour Behavioural experiments Action planning Prompts/cues	Advise GPs to record the outcomes of consultations involving self‐harm, to compare actions taken, or risk assessments made, with what is recommended in the guidelines (IF: Training; BCT: Self‐monitoring outcomes of behaviour).
Physical opportunity	Environmental context and resources	Guidelines are perceived to be inaccessible, and do not account for restricted time and lack of secondary resources available.	‘There's so much that we can do, but often we need secondary care to help, or, you know, other people to be involved, because, at the end of the day, we see a patient for 10 or 15 minutes and then they're gone and they're having to wait for a referral or other services’ (Salaried GP 1)	Environmental restructuring Enablement	Restructuring the physical environment Restructuring the social environment Action planning	Prompt GPs to plan how they will respond to patients who are below the threshold for immediate suicide risk when referrals are unavailable (IF: Enablement; BCT: Action planning).
Reflective motivation	Beliefs about capabilities	Guidelines provide reassurance about GP’s role in managing self‐harm. Challenging to monitor patients waiting for referrals.	‘I don't do things that any other GP doesn't do, yes? What I’m describing to you is best practice and there will be days I can't meet best practice but I will at least meet safe, good enough, effective practice, yes?’ (Salaried GP 12)	Education Persuasion Enablement	Verbal persuasion about capability Focus on past success Action planning	Advise GPs to describe occasions where they implemented the national guidelines with a patient to reassure them about their judgement (IF: Enablement; BCT: Focus on past success).

Note

No BCTs are associated with ‘memory, attention, and decision processes’, so BCTs were associated with the intervention functions.

Abbreviations: BCT, Behaviour change technique; IF, Intervention Function.

Figure 1 demonstrates considerable convergence between explanatory themes; three groups were perceived to consist of both enablers and barriers, related to information and skill needs for challenging consultations, guideline engagement and clinical uncertainty surrounding diagnosis and long‐term care for self‐harm. Despite this overlap, the specific concepts within each theme are domain‐specific and were coded as either an enabler or barrier depending on the GPs’ descriptions.

Five theoretical domains emerged that explained the barriers and enablers to implementing the NICE guidelines for self‐harm: *knowledge* (reported by 62% of GPs); *cognitive and interpersonal skills* (reported by 86% of GPs); *memory*, *attention and decision processes* (reported by 67% of GPs); *environmental context and resources* (reported by 100% of GPs); and *beliefs about capabilities* (reported by 67% of GPs). Explanatory quotes with participant ID are displayed in parentheses.

### Knowledge

#### Broad principles of mental health risk assessment (enabler)

Twelve participants reported being trained as junior doctors to conduct general mental health risk assessments in line with NICE guidance, which included being capable of identifying self‐harm as a risk factor for suicide. They utilize these same principles of risk assessment with all mental health presentations, including those that involve self‐harm. This feature of training was reported across current trainees and longer practising locum and salaried GPs.

Within GP training, having that formal bar of, ‘This is what you do within a self‐harm/suicide risk/mental health consultation,’ is useful to have… So, not everybody will practice the way that I do, that was just the way that I was trained and probably the time that I was trained is always think of what the red flags are, find out those at the consultation. (Trainee GP 20)

#### Enhanced self‐harm training (enabler)

Seven participants described their knowledge about self‐harm as poor following GP training, which led them to seek out more training after qualifying. Others mentioned having greater capacity to respond to self‐harm because of rotations in psychiatry or emergency services as a trainee. Participants highlighted the value of learning from case studies and patient‐led training to inform GPs of risk cues and how to ask salient questions. This manner of learning was considered superior to lectures or online training modules, since it directly enhances GPs’ capabilities by defining prompts and cues (BCTTv1 7.1) in an applied manner. This enabler suggests the existing knowledge about self‐harm among trainee GPs may require improvement through Education interventions.

I’ve done face‐to‐face training within the last couple of years… that was actually led by a survivor that was available through the local NHS health department… If you are in healthcare, every single member should have access to that type of training, because if you do not pick up the cue, you do not recognise that there is even a potential for risk and that is when people fall through the net. (Salaried GP 12)

### Cognitive and interpersonal skills

#### Skilled communication a necessary part of GP role (enabler)

Communication skills were described by 18 participants as an integral part of a GP’s role and necessary to gather information about a patient quickly and sensitively. GPs draw on these skills when assessing risk following self‐harm by looking and listening for risk cues and by reassuring patients that self‐harm is a common feature among people who experience low mood or depression. Effective communication strategies were developed through a combination of training and face‐to‐face experience; there was a pervasive perception that some aspects of communication (e.g. question wording) could be taught formally through Training interventions, but personality and experiential learning in the consulting room were overriding factors to assessment styles.

I think that's completely key when you're doing work on mental health problems, because they may well feel ashamed, they might well feel really uncomfortable about what's going on, they might not understand it themselves, they might not be able to put it into words clearly… So you have to be able to kind of listen to whatever it is that they're saying, and then try and help them to give you enough information for you to be able to support them and find the things that are going to help them. (Trainee GP 5)

#### Difficult to remain sensitive when under pressure (barrier)

Participants described complications conducting risk assessments with distressed patients, especially within the context of a standard ten‐minute appointment. GPs sometimes have to overcome patients’ reluctance to discuss self‐harm and reported difficulties making patients feel comfortable. To navigate this barrier, GPs often encourage patients to return for multiple appointments to build a rapport over time and make better judgements about risk.

I think a lot of patients would struggle to open up, and particularly with the time management I think some doctors would be trying to hurry the patient along, and I think strategies to deal with patients compassionately but efficiently would be helpful, because we need to get a certain amount of information out of patients in order to do these risk assessments and that can be quite difficult to do sensitively in the time that we're allowed. (Locum GP 16)

### Memory, attention and decision processes

#### Perceived distractions from patient cues (barrier)

Participants were reluctant to refer to self‐harm guidance during consultations in case they missed an important cue from the patient, or disrupted the process of rapport building. GPs tend to utilize the flow of conversation to naturally gather information instead of asking risk assessment questions by rote. There were concerns among participants that they would be required to go through a set of ‘tick‐box’ questions with patients to accurately implement the NICE guidelines.

Part of me feels like you could lose a little bit of patient, you know, that empathy… Yes, to say ‘Right, now, here's another form and I’d like to ask you these and let's tick the boxes,’ and you can sometimes lose the connection you've got with the patient by doing that… Sometimes just a free‐flowing conversation you can get more information maybe, if you're asking the right questions. (Salaried GP 1)

#### Professional judgement and patient history (barrier)

Previous experience and ‘gut feelings’ about risk took precedent in participants’ decision‐making; as a result, GPs did not feel the need to check national guidance to inform their consultations. GPs tended to trust that their professional judgement was consistent with the expectations of the NICE guidelines. Six participants reflected on whether their practice was in line with the guidance, and three mentioned concerns that deviating from them could result in punishment or litigation if a patient subsequently died by suicide. However, participants emphasized that urgent self‐harm presentations are rare; more often than not, they made decisions about longer term care based on underlying mental health issues. As a result, they did not necessarily take the time to focus on self‐harm behaviour in isolation because establishing a patients’ risk of suicide eclipses the need to establish risk of non‐suicidal self‐harm.

But then that's also the downside of then you're taking‐, a lot has to be said for, like, gut‐feeling in these decisions. A lot goes for, as a GP, your familiarity and your knowledge of the patient. Not all of your patients, but a lot of your patients that you see regularly, you know them on a different level to other services because you see them more regularly. (Trainee GP 4)

#### Screen alerts provide prompts (enabler)

Internal communication platforms such as TeamNet are commonplace in practices and are used by managers to disseminate information to their staff quickly. Participants who received online alerts from such systems whenever new guidance is released or old guidelines are updated found this to be an efficient way to stay updated about guideline content (BCTTv1 7.1: Prompts/cues). However, older guidance that has not changed, including the self‐harm guidance at the time of writing, is not routinely circulated.

I’m kind of registered with the NICE guidelines online, so I get an alert when there's a new guideline or kind of an updated guideline, so I’ll have a look if I get one of those alerts. (Locum GP 9)

### Environmental context and resources

#### Lack of secondary resources (barrier)

Participants provided several examples of frustrating circumstances when trying to refer self‐harm patients to a specialist mental health service and reported discrepancies between the guidelines’ appropriate reasons to refer and secondary services’ criteria for accepting a patient. Crisis teams and secondary mental health services were described as being overloaded and difficult to reach, so GPs were often unable to make referrals commensurate with the expectations of national guidelines unless a patient was at immediate risk of suicide. GPs also noted that they frequently had no local third‐sector alternatives, leaving patients below the threshold for immediate suicide risk needing to be managed in their care.

I feel like we go around in circles a lot. ‘This patient is not going to kill themselves, discharge back to the GP,’ or, ‘Have some diazepam, discharge back to the GP,’ which then creates another issue… Sometimes they can be brilliant, and sometimes they can get other services involved, or refer to self‐harm services that we didn't even realise were available, or organise counselling and things like that. Sometimes, it's just a constant circle of feeling like we're getting nowhere. (Trainee GP 4)

#### Guideline content perceived to be vague and unrealistic (barrier)

The presentation of the guidelines was identified as a barrier; some found the appearance and wording of the NICE guidance too long‐winded and off‐putting. Participants criticized the layout of the NICE guidance website as being difficult to navigate, which made them unsuitable for quick reference when in consultation with a patient. Additionally, some participants found the guidance for GPs was vague, with an unrealistic reliance on the availability of secondary services.

The trouble with these guidelines is that if it doesn't give you good and clear information about what kind of things you can do for someone, you end up with a lot of words that often don't mean anything and people are lot less likely to look at them. I’m certainly not going to sit and read absolutely all of these statements because I don't have time and I don't have the inclination to do it. I think you need more bullet points and easier to understand and follow, otherwise people aren't going to read them. (Partner GP 2)

#### Time constraints (barrier)

A fundamental barrier in general practice is appointment length. Participants felt it unrealistic to expect a complete risk and needs assessment within ten minutes and mentioned that double‐ or triple‐length mental health appointments were common. Support systems exist in some practices to facilitate this by shuffling appointments between other practice staff, providing GPs with more time to assess their patient (BCTTv1 12.1: Restructuring the physical environment). However, extended appointments come at the cost of other patients’ and colleague workloads; additionally, such systems are ultimately unfeasible to enact in solo or remote practices.

You're supposed to do all this, sort of, detailed assessment with the patient in ten minutes. It doesn't happen. If you have a good‐going depression case with lots of risk factors, you're there 20–30 minutes with the patient… You don't always see that extent of patients who need that amount of input every single day. So, if it's happening once or twice a week then, you know, it's doable. Yes, it makes you run a bit late for your other patients, but at least it means, well, you've done the job properly. (Trainee GP 3)

### Beliefs about capabilities

#### Guidelines provide confidence and reassurance (enabler)

Since acute self‐harm is a relatively rare occurrence in primary care, the guidelines provide a foundation for a GP’s response depending on the nature of the presentation and the perceived urgency to act. Although longer practising GPs felt more confident relying on their professional judgement, trainees reported referring to the guidelines to reassure both themselves and their patient that they are making evidence‐based decisions. Some participants reported relief that the guideline did not contain actions they were not already implementing. While more experienced GPs criticized the guidelines for being too ‘commonsense‐ical’ (Locum GP 21), they also believed the guidance was well within the capabilities of any GP.

You've got to be able to give the patient a sense that… you know what you're doing. They've come to see you at their worst moment. You've got to be able to say; look, I can help you, and I can do that if I know that I’m not just drawing on rubbish or not just drawing on my own, sort of, you know, random little memory somewhere. I’ve got something here that I’ve got evidence for. (Trainee GP 11)

#### Uncertainty about remit when monitoring a patient waiting for referral (barrier)

GPs described being the first port of call for many patients who have mental health difficulties and consider themselves to have a dual role to signpost patients towards appropriate services, in addition to providing validation and a listening ear. However, the time a patient will spend waiting for a referral following self‐harm varies, which leaves GPs with an uncertain outlook about how best to manage the patient in the meantime. The guidelines do not currently detail how GPs should monitor patients while they are waiting; some participants argued that as generalists, they are not well placed to care for patients with mental health issues long‐term, and it is unfair that they are expected to take on the roles of mental health professionals in the absence of specialist resources. GPs mentioned that it is unfeasible in busy practices to create repeat appointments to monitor patients regularly.

I think the main thing that would be useful for GPs is to have some sort of strategy about how to treat these patients before they actually wait for ages to see a psychologist, so I think we're all very aware that referring for counselling is important but that doesn't necessarily help patients because if they're waiting for three months to see someone, by the time they've waited three months to see anyone they've got themselves into a right state. (Salaried GP 8)

### Intervention development: Proposed functions and exemplar BCTs

Exemplar interventions, domain descriptions and exemplar quotes are presented in Table 2 to address the second aim of this research; intervention functions and BCTs were mapped according to the Behaviour Change Wheel. Five of nine intervention functions (Michie et al., 2011) were linked to five TDF domains: education, training, environmental restructuring, enablement and persuasion. Eight of sixteen BCT groupings were found to be relevant: feedback and monitoring, shaping knowledge, natural consequences, associations, repetition and substitutions, antecedents, goals and planning, and self‐belief. Fourteen unique BCTs were found to be relevant. For example, to target *cognitive and interpersonal skills* interventions might comprise: prompting GPs to practice de‐escalating a distressed consultation through role‐play exercises with colleagues (intervention function: Training; BCT: Behavioural rehearsal/practice), or by encouraging GPs to regularly check for new or updated NICE guidance online (intervention function: Training; BCT: habit formation).

## DISCUSSION

### Main findings

This is the first study to use a theoretically grounded framework to identify barriers and enablers that influence GPs’ implementation of the NICE guidelines for self‐harm. The framework (Atkins et al., 2017) examined how these influences could inform the development of an intervention to support guideline implementation in primary care. This study contributes to existing literature by identifying five distinct TDF domains that illuminate and encapsulate the challenges GPs face to implementing national guidelines for self‐harm. Three broad targets for intervention were identified from the explanatory themes: information and skill needs, guideline engagement and clinical uncertainty. We provide recommendations for relevant intervention functions and BCTs that could be incorporated into quality improvement interventions to empower GPs to implement NICE guidelines when they encounter a patient seeking help for self‐harm.

In terms of information and skill needs, existing research corroborates that GPs feel under‐skilled to discuss self‐harm (Bailey et al., 2017; Fox et al., 2015) (*Environmental context and resources*) and struggle to conduct brief, empathetic consultations when discussing emotional concerns due to the time and temperament needed to de‐escalate distress (Parker et al., 2020) (*Cognitive and interpersonal skills*). Numerous studies have identified knowledge gaps among GPs about self‐harm (Cello Health PLC, 2012; Mughal et al., 2020), particularly in relation to recognizing self‐harm as a risk factor for suicide (Fox et al., 2015) (*Knowledge*). The Royal College of General Practitioners’ (RCGP) curriculum for GP training recognizes self‐harm as a ‘common and important condition’, but the material provided to trainees about self‐harm remains limited (Royal College of General Practitioners, 2019). Furthermore, information about self‐harm is obscured in the RGCP’s Mental Health Toolkit for GPs under ‘Suicide and Crisis Care’ (Royal College of General Practitioners, 2021). While knowledge and skill gaps could be addressed through enhanced education, GPs emphasized that receiving information about self‐harm directly from patient experts by experience provides practical feedback and information about health consequences (Dijk et al., 2020).

GPs rarely spontaneously engaged with guidelines during consultations due to perceptions that they could distract from patient cues (*Memory*, *attention and decision processes*) (Parker et al., 2020). Environmental pressures such as time constraints and a lack of specialist mental health resources are common obstructions to conducting assessments and arranging referrals (Bailey et al., 2019; Bruco et al., 2018) (*Environmental context and resources*). Additionally, guideline length and complexity can discourage busy GPs from taking the time to read them (Francke et al., 2008). Optimization of NICE guidelines for quick reference could help to address engagement issues raised by GPs about the readability of the guidance. Electronic prompts were praised as an enabler by GPs; however, research suggests prompts alone do little to improve implementation (Brennan et al., 2018); technological interventions must also consider broader system issues within the dynamic contexts of primary care (Keyworth et al., 2018; Litchfield et al., 2018).

While some GPs felt reassured that the guideline content contained common‐sense recommendations within their skillset, particularly in relation to mental health assessments (*Beliefs about capabilities*), others held more sceptical attitudes about whether the guidance was within their remit as generalists; similar criticisms have been made by GPs about the utility and trustworthiness of guideline content (Carlsen et al., 2011; Carlsen & Norheim, 2008). Most notably, GPs reported uncertainty about how best to monitor patients on waiting lists for specialist care, making decisions based on their prior knowledge of the patient and professional judgement. It is common for healthcare professionals to rely on professional judgement over clinical guidelines when addressing self‐harm (Mughal et al., 2022), due to disagreements with the guideline content and beliefs about patients’ needs (Austad et al., 2015; Francke et al., 2008). Therefore, the lack of clarity in the guidelines about what GPs should do in terms of monitoring and follow‐up prevents them from engaging with the guidelines and may risk creating unrealistic expectations about the care non‐mental health professionals are able to provide for such complex behaviour.

Automatic motivation and physical capability are notable by their absence from our analysis. Although reinforcement and emotional drivers were reported by nine and four of the participants respectively, they were not mentioned sufficiently to warrant inclusion according to our criteria. Considering the association of automatic motivation with implementation of the NICE guidelines for self‐harm (Leather, J. Z., Kapur, N., Campbell, S. M. & Armitage, C. J., unpublished data) and its role in guideline implementation in other areas of practice (Egerton et al., 2018; Kredo et al., 2018), it is surprising that habitual and emotional processes were omitted from most responses. This may be due to an inability to articulate the influence of higher level automatic processes (e.g. habit) on behaviour (Nisbett & Wilson, 1977). Alternatively, it may demonstrate that most GPs have not yet developed a habit of implementing self‐harm guidelines; which would provide an opportunity for interventions to replace old habits with guideline consistent behaviours (Cottrell et al., 2016; Egerton et al., 2018). The non‐inclusion of physical capability in participants’ accounts may reflect the limited role of physical exertion or fine motor skills in general practice compared to other clinical roles (Ierano et al., 2019); or that GPs believe they are capable of the tasks involved in their role (e.g. physical examinations).

### Implications for practice, implementation and policy

**FIGURE 2:**
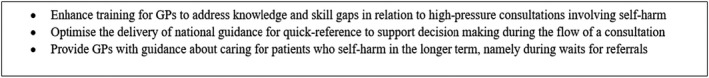
Summary of implications for practice, implementation and policy

This study has identified several areas where GPs require support to implement the NICE guidelines for self‐harm during routine consultations, in addition to suggestions to address deficiencies in the accessibility of the guidance (summarized in Figure 2). First, GPs require enhanced training to remediate knowledge and skill gaps in relation to self‐harm risk assessments, particularly during high‐pressure consultations. Second, the guidelines need to be optimized for quick reference to support decision‐making in a way that maintains the flow of consultations. Lastly, more clarity and detail must be provided in relation to long‐term management in primary care, providing it is realistic for busy GPs to implement. The Mental Health Toolkit (Royal College of General Practitioners, 2021) represents an opportunity to deliver information needs about self‐harm in a timely and accessible manner. The implementation of NICE guidelines for self‐harm could be a candidate area for quality improvement in future iterations of the NHS Quality and Outcomes Framework.

We have provided recommendations about specific BCTs to utilize in future interventions, which are derived from TDF domains that emerged from participant responses (represented in Table 2). Exemplar interventions from the analysis could include providing information about patterns of risk identifiable in patient notes prior to consultations (Intervention function: Education; BCT: Information about antecedents); developing a short‐form version of the guidance for GPs (Intervention function: Environmental restructuring; BCT: Restructuring the physical environment); using implementation intentions to provide GPs with if‐then responses to common consultation scenarios (Intervention function: Enablement; BCT: Action planning). Pilot interventions would require feasibility and acceptability testing with involvement from GPs to develop realistic and impactful improvements to guideline implementation.

### Strengths and limitations

Utilization of the TDF and BCT Taxonomy V1 (Michie et al., 2013) has provided a robust foundation for future research and intervention development. The findings represent a synthesis of information corroborating existing findings from disparate studies into a single, comprehensive series of recommendations. A strength of basing the interview schedule on the COM‐B instead of the TDF is that participants could naturally report on barriers and enablers, which created opportunities to conceptualize non‐TDF drivers (McGowan et al., 2020).

However, there were some limitations. The sampling frame recruited GPs with varying career lengths; however, there was an over‐representation of trainee and newly qualified doctors whose perspectives could be limited by their lack of experience (Shiner & Howe, 2015). Additionally, due to the nature of follow‐up research, we were unable to expand our sampling frame to rectify the gender gap in this sample compared to General Practice Workforce statistics (NHS Digital, 2022); including the experiences of more male GPs than were included in the present study may have afforded prominence to different themes or domains in the data. Explicitly deriving interview questions from TDF constructs may have prompted more focused discussion from participants about specific barriers and enablers. On the other hand, the spontaneous emergence of TDF‐congruent codes and themes supports the validity of Behaviour Change Wheel constructs mapping onto the TDF (Atkins et al., 2017). The criteria used to select relevant domains may have resulted in important domains being overlooked, either because they were mentioned infrequently or because they were not emphasized as important by participants. Alternative analytical approaches such as grounded theory may reveal additional barriers and enablers not sufficiently explained by the TDF framework (Mosavianpour et al., 2016).

## CONCLUSION

GPs have a multifaceted role in assessing and managing self‐harm and require a range of support mechanisms to implement national guidelines for self‐harm. Utilizing the Behaviour Change Wheel has (1) identified barriers and enablers that GPs face to implementing the NICE guidelines for self‐harm and (2) provided exemplar intervention strategies derived from TDF domains and relevant BCTs. The five domains highlighted in this study could be targeted individually or together in complex quality improvement interventions. Given the volume of self‐harm presentations in primary care and prevalence of self‐harm in the United Kingdom, these findings provide timely recommendations to support GPs to assess and manage self‐harm.

## CONFLICT OF INTERESTS

JZL reports grants from NIHR PSTRC; CJA was supported by NIHR Manchester Biomedical Research Centre; NK was supported by the Greater Manchester Mental Health NHS Foundation Trust. NK chaired the NICE guideline development group for the long‐term management of self‐harm and the NICE Topic Expert Group (which developed the quality standards for self‐harm services). He is currently chair of the updated NICE guideline for Depression and Topic Advisor to the new NICE self‐harm guideline. NK is a member of the Department of Health's (England) National Suicide Prevention Advisory Group and works with NHS England on national quality improvement initiatives for suicide and self‐harm. The views expressed in this article are the authors’ own and not those of the NIHR, Department of Health and Social Care NHS England or NICE.

## AUTHOR CONTRIBUTION

**Jessica Z Leather:** Data curation; Formal analysis; Project administration; Visualization; Writing – original draft. **Christopher Keyworth:** Formal analysis; Writing – review & editing. **Nav Kapur:** Supervision; Writing – review & editing. **Stephen M. Campbell:** Supervision; Writing – review & editing. **Christopher J. Armitage:** Conceptualization; Funding acquisition; Project administration; Resources; Supervision; Writing – review & editing.

## Data Availability

Data available on request from the authors.
