# Supplementary material for: Examining drivers of self‐harm guideline implementation by general practitioners: A qualitative analysis using the theoretical domains framework
Source: Br J Health Psychol. 2022 Apr 13;27(4):1275–95. doi: 10.1111/bjhp.12598 (PMC9790562; doi:10.1111/bjhp.12598)
Supplement: Supplementary file 1 [file BJHP-27-1275-s002.docx]

**Interview Topic Guide**

| **Question** | **Construct (if relevant)** |
| --- | --- |
| **Your role** | |
| 1. Talk me through your typical working day |  |
| 1. What role does a *<health professional>* play in assessing or managing patients who are at risk of self-harm?  - Have you ever encountered a patient you thought was at risk of self-harm? - How often do you typically encounter patients who are at risk of self-harm? |  |
| 1. During your time as a *<health professional>*, how has the assessment and management of self-harm in your practice/place of work changed?  - Have protocols changed over time? |  |
| 1. Can you tell me about any training you have received for assessing or managing self-harm?  - Did you find it useful for encountering patients at risk of self-harm? - Is there anything you would change about your training?   *If no training*: What would you expect from training to prepare you for encounters with patients at risk of self-harm? |  |
| 1. Are you aware of any tools you might use when encountering a patient who is at risk of self-harm?  - What is the tool? How would it help you? - Could it be improved?   *If no tools used*: Is there anything you can think of that would help you to assess and manage a patient at risk of self-harm? |  |
| 1. Do you see self-harm prevention as something you are personally responsible for?  - To what extent is it a priority for you? |  |
| 1. Talk me through what you would do if you encountered a patient who you thought was ***at risk*** of self-harm.  - If relevant, you can describe a past encounter |  |
| 1. Now tell me about what you would do if you encountered a patient who had ***actually*** self-harmed.  - If relevant, you can describe a past encounter |  |
| 1. Can you tell me about what you would do if you thought you needed to refer a patient who was at risk of self-harm, either for a psychosocial ***assessment*** or for a psychosocial ***intervention***? (*e.g. cognitive behavioural therapy*)  - Would you expect to encounter any difficulties with the process? |  |
| **General perceptions of the NICE guidelines for assessing and managing self-harm** | |
| 1. What do you think about the NICE guidelines for assessing and managing self-harm ***in general***?  - Do you think they are suitable? Why/why not? | Reflective motivation |
| 1. What do you think about implementing the NICE guidelines for self-harm ***in your role/place of work***?  - Do you think they are suitable? Why/why not? - How easy is it for you to implement the NICE guidelines? | Reflective motivation |
| 1. What do you think are the benefits of implementing the NICE guidelines for assessing and managing self-harm?  - To patients - To you personally |  |
| **Role responsibility for implementing NICE guidelines for self-harm** | |
| 1. Would you say that generally you are in a habit of implementing the NICE guidelines for self-harm?  - What would be helpful in developing a routine/habit of implementing the NICE guidelines? | Automatic motivation |
| 1. What skills do you think are required to implement the NICE guidelines for self-harm?    - To what extent do you have these skills?    - How did you acquire these skills (training or experience)? | Physical and Psychological capability |
| 1. Do you receive any support from your colleagues to implement the NICE guidelines for self-harm?  - Are the guidelines promoted in your workplace? | Social opportunity |
| 1. Do you think your colleagues implement the NICE guidelines for self-harm? | Social opportunity |
| **Suggestions** | |
| 1. To what extent does your work environment provide the opportunity to implement the NICE guidelines for self-harm?  - What would need to change about your workplace for you to better implement the guidelines? | Physical opportunity |
| 1. What are the main challenges of implementing the NICE guidelines for self-harm with patients?  - Is there anything specific that prevents you from implementing the guidelines? |  |
| 1. What, if anything, would make implementing guidelines about assessing and managing self-harm easier for you as a *<health professional>?* |  |
| 1. Is there anything that we haven’t covered that you feel is important/relevant? |  |

*COM-B questions developed according to Michie et al. (2014)
